# Deep learning for high-resolution and high-sensitivity interferometric phase contrast imaging

**DOI:** 10.1038/s41598-020-66690-7

**Published:** 2020-06-18

**Authors:** Seho Lee, Ohsung Oh, Youngju Kim, Daeseung Kim, Daniel S. Hussey, Ge Wang, Seung Wook Lee

**Affiliations:** 1 School of Mechanical Engineering, Pusan National University, Busan, 46241 Republic of Korea; 2 Neutron Physics Group, National Institute of Standards and Technology, Gaithersburg, MD 20899 USA; 3 Department of Biomedical Engineering, Rensselaer Polytechnic Institute, Troy, NY 12180 USA

**Keywords:** Imaging and sensing, Imaging techniques

## Abstract

In Talbot-Lau interferometry, the sample position yielding the highest phase sensitivity suffers from strong geometric blur. This trade-off between phase-sensitivity and spatial resolution is a fundamental challenge in such interferometric imaging applications with either neutron or conventional x-ray sources due to their relatively large beam-defining apertures or focal spots. In this study, a deep learning method is introduced to estimate a high phase-sensitive and high spatial resolution image from a trained neural network to attempt to avoid the trade-off for both high phase-sensitivity and high resolution. To realize this, the training data sets of the differential phase contrast images at a pair of sample positions, one of which is close to the phase grating and the other close to the detector, are numerically generated and are used as the inputs for the training data set of a generative adversarial network. The trained network has been applied to the real experimental data sets from a neutron grating interferometer and we have obtained improved images both in phase-sensitivity and spatial resolution.

## Introduction

**Figure 1:**
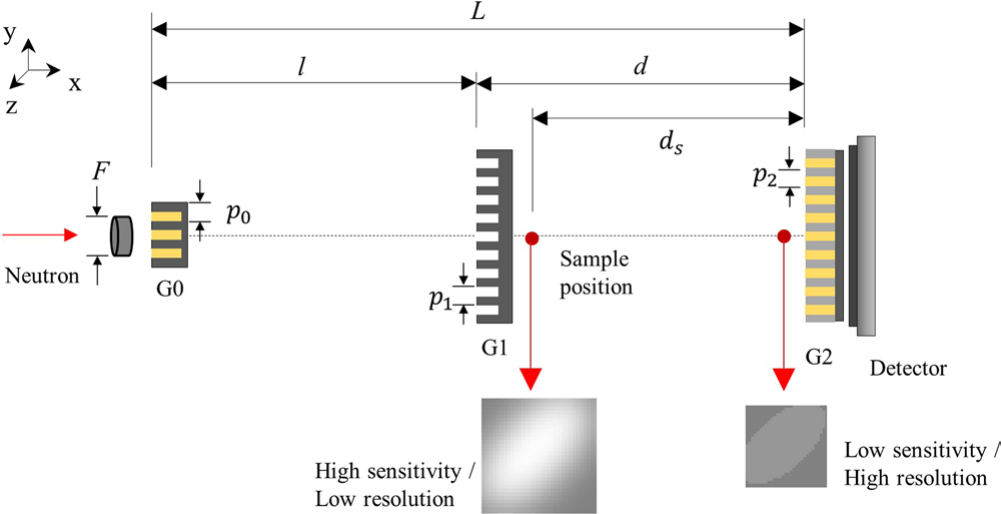
Schematic of the Talbot-Lau grating interferometer of neutron.

The Talbot-Lau grating interferometer is a promising technology for phase contrast imaging. Importantly for neutron and conventional X-ray sources, Talbot-Lau interferometry is effective for an incoherent beam through the use of a source grating that imparts the required quasi-coherence^1,2^ and is widely adopted for use with these radiation sources. The Talbot-Lau interferometer is composed of three gratings: source, phase, and analyzer. Figure 1 shows a schematic of the neutron Talbot-Lau interferometer. The source grating G0 imparts transverse coherence to the initially incoherent beam. The phase grating G1, modulates the phase of the wave by π, creating a near-field diffraction pattern known as a Talbot carpet. This pattern is slightly deformed by a sample and resolved by an analyzer transmission grating G2. The Talbot-Lau interferometer is normally classified into three kinds of geometry according to the distance between each grating: conventional, symmetric, and inverse. The conventional geometry is a common configuration in neutron and X-ray implementations, and the distance *l* (G0 to G1 separation) is longer than *d* (G1 to G2 separation). The inverse geometry is vice versa configuration with the conventional geometry. When the distance *l* = *d*, the Talbot Lau interferometer is in the symmetric geometry.

Donath *et al*.^3^ derived the phase sensitivity by means of the distance *d*, sample position *d*_*s*_, period of the G2 grating p_2_ and so on. From this derivation, the maximum phase sensitivity is generally satisfied under two conditions^4–6^. The first is the symmetric configuration^4^, and the second is to position the sample close to the phase grating G1^5,6^. Since the sensitivity of the phase contrast imaging is generally related to the image contrast, it is important to design a high-sensitivity system for better performance. Despite the high phase-sensitivity in the symmetric geometry, this configuration results in image blurring due to the large distance between the detector and sample. The phase-sensitivity/spatial resolution trade-off problem is severe when the radiation source has a large focal spot size or beam defining aperture, such is the case with conventional x-ray tubes and neutron beams^7,8^. In addition, the high sensitivity image causes image distortion in the region where the phase gradient is large^8^. In this case, it is still important to acquire high-sensitivity information even if a certain area is lost, because higher sensitivity can increase the contrast in regions with smaller gradients. Therefore, studies on how to correct these problems will be of great significance for application in medical and industrial fields.

This paper introduces a method to obtain a corrected image which has high phase-sensitivity as well as high spatial resolution (hereafter referred to simply as sensitivity and resolution, respectively) for a symmetric Talbot Lau interferometer using a deep learning technique. We acquire two images, one with the sample positioned close to the phase grating G1 (high-sensitivity), and another with the sample positioned in front of the analyzer grating G2 (high-resolution). We then trained a neural network^9^ to address the sensitivity-resolution trade off problem for our experimental setup.

To train the neural network, the high-resolution and high-sensitivity phase contrast image should be required as ground-truth. Acquiring a phase contrast image with high resolution and high sensitivity in an experiment is challenging, so this study designed a creation system to imitate pseudo-phase contrast image with general color pictures. In here, we considered the phase contrast image obtained from the grating interferometer is a gray scale image normalized in the range of −π to + π. We then converted the RGB 3-channel matrix to a 1-channel matrix and normalized from −π to + π to mimic the phase contrast images. The blurring and contrast level are additionally added to imitate phase contrast images in the two different positions. From the idea, we expect to train neural network to produce optimal results from two different inputs.

Traditional neural networks to improve image quality are convolutional neural network (CNN) and generative adversarial network (GAN). One resolution improvement technique known as super-resolution CNN (SRCNN) estimates unsharpness through the trained neural network and restores the resolution of the blurred input image^8,10–18^. As CPU and GPU technologies develop, very deep convolution layers of neural networks can be calculated. Kim *et al*.^14^ reported very deep super resolution CNN (VDSR), and verified much better performance with 20 convolution layers. This CNN, which has excellent performance, is now applicable in a wide range of applications such as medical diagnosis, monitoring service, precision analysis, and other computer vision tasks^19–29^. Another architecture for resolution improvement is the GAN, a deep residual network that has also shown excellent results^9^.

However, the problem addressed in this study is somewhat different from the resolution improvement study. The high sensitivity phase contrast image causes irregular image distortion in regions where there is considerably high phase-shift, and this error is not easy to correct perfectly. Instead of acquiring a single image in the compromising position, this study attempts to resolve this problem by matching two images. We built a neural network with two input channels to extract information of high sensitivity and high resolution, and finally reconstructed a phase contrast image with both high resolution and high sensitivity. Our network is described in the Methods section under Deep Learning Network.

## Results

**Figure 2:**
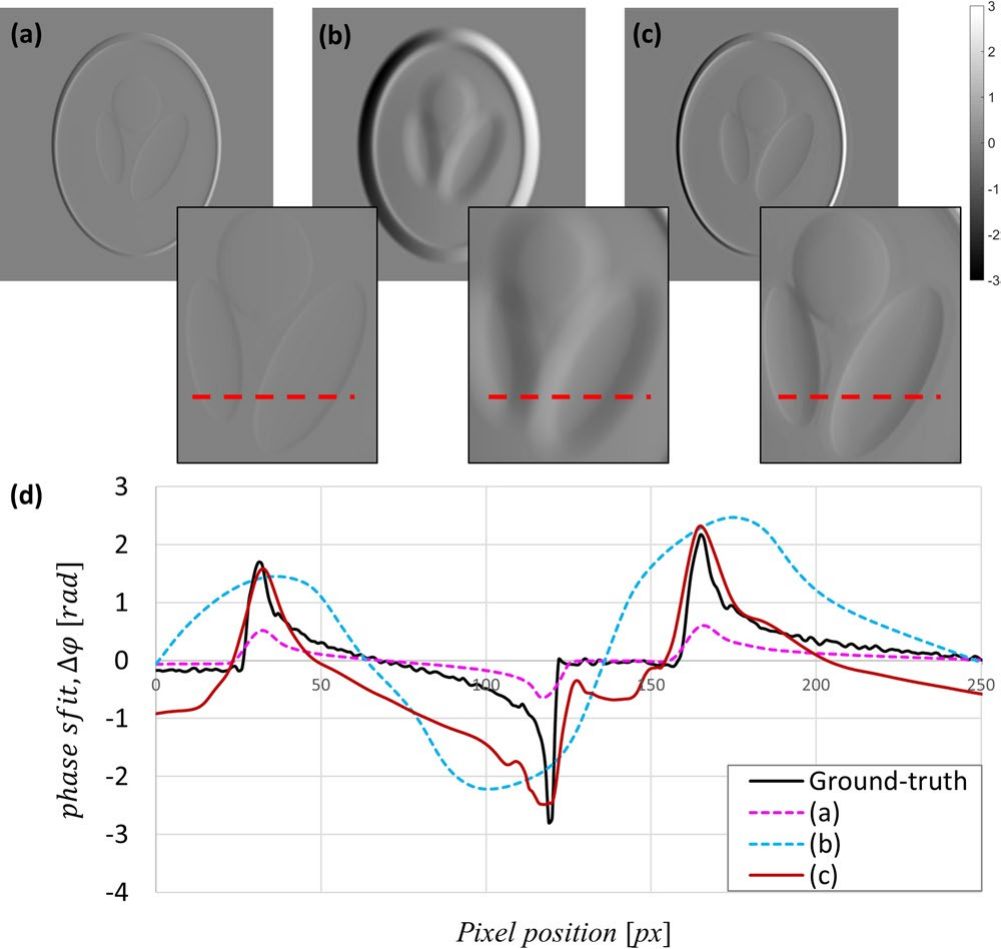
Simulation results using the Shepp-Logan phantom. (**a**) is the low-contrast high-resolution image, and (**b**) is the high-contrast low-resolution image. The GAN result combined with (**a**) and (**b**) is shown in (**c**). (**d**) shows the profile of the lines in (**a**), (**b**), and (**c**) with the ground-truth. Our 2-input GAN profile in red shows a good agreement with the ground truth in black.

A simulation was conducted to evaluate our study. As inputs of our network, a low-contrast high-resolution image and a high-contrast low-resolution image are required. To prepare inputs, a 3-D Shepp- Logan phantom was numerically created, and a projection image was acquired using ASTRA Toolbox^30,31^. A differential phase contrast image was imitated by generating a differential image of the acquired projection image. The differential image presented in Fig. 2(a) was defined as a low-contrast, high-resolution image. The high-contrast low-resolution image presented in Fig. 2(b) was created by multiplying a constant to Fig. 2(a) and inducing a gaussian filter to make it blurred. In the neutron measurements, the sensitivity is linear in the sample position, so the sensitivity of these numerical studies can be mimicked by a linear scaling of the differential image. The resultant image corrected by our GAN model is in Fig. 2(c). Figure 2 (d) shows the profile of each input image and the GAN result and shows that the two input images Fig. 2(a,b) are properly combined and represents a better estimate of the ground truth than either of the input images.

**Figure 3:**
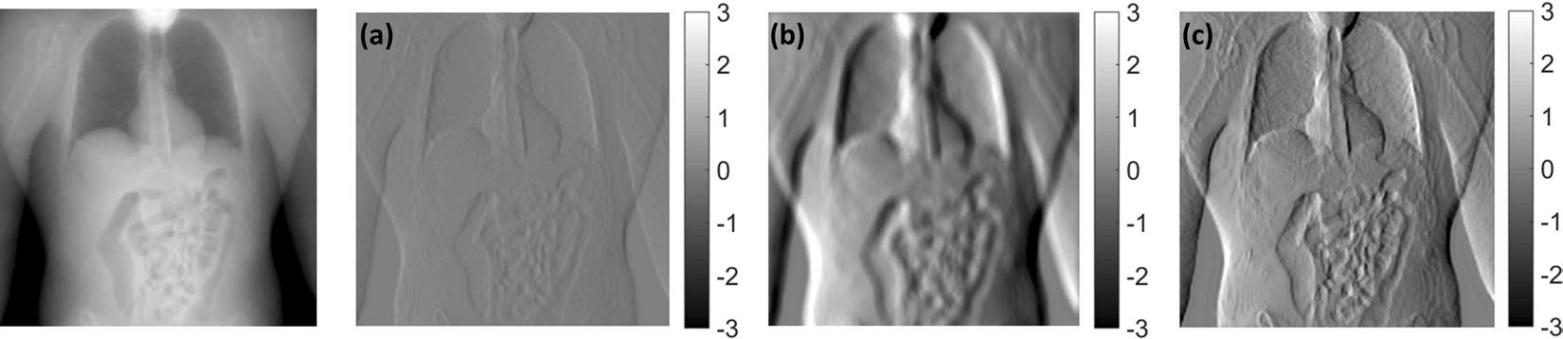
Simulation results using XCAT clinical phantom, shown at the left without a label. (**a**) and (**b**) are the low-contrast high-resolution image and the high-contrast low-resolution image, respectively. (**c**) is GAN result. The result is well combined by (**a**) and (**b**).

For a more complex shape, the clinical phantom was also created by XCAT^32^. Figure 3(a,b) are the low-contrast high-resolution and high contrast low-resolution images, respectively. The GAN result shown in Fig. 3(c) shows that two images are well combined even in the complex shapes.

**Figure 4:**
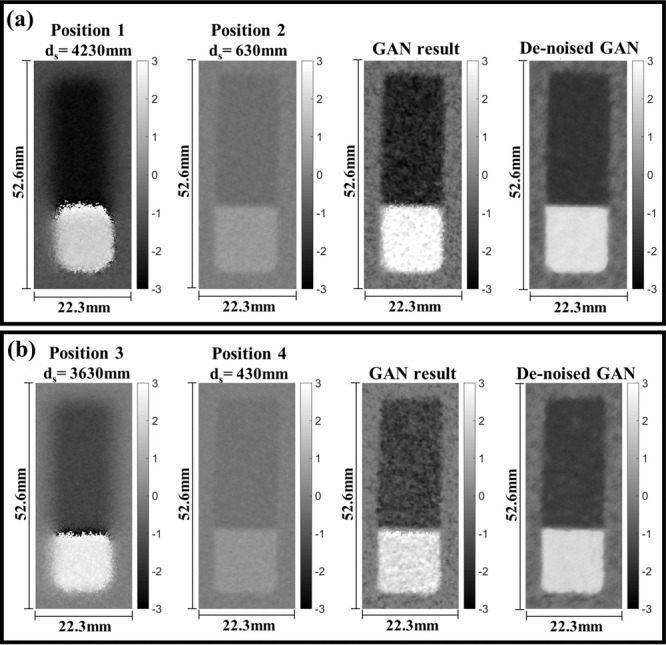
The two different pairs, that is, (**a,b**) show the results of our study. Each pair shows two input images and corrected results of a Si wedge. In each image, the dark rectangle represents the angle of 27° and the bright rectangle represents the angle of 46°. The sample positions (d_s_) for (**a**) are 4230 mm and 630 mm, and those (**b**) are the 3630 mm and 430 mm. Position 1 in (**a**) and 3 in (**b**) represent high-sensitivity, and position 2 in (**a**) and 4 in (**b**) represent high-resolution images. The corrected results are shown as ‘GAN result’ and ‘De-noised GAN’.

**Figure 5:**
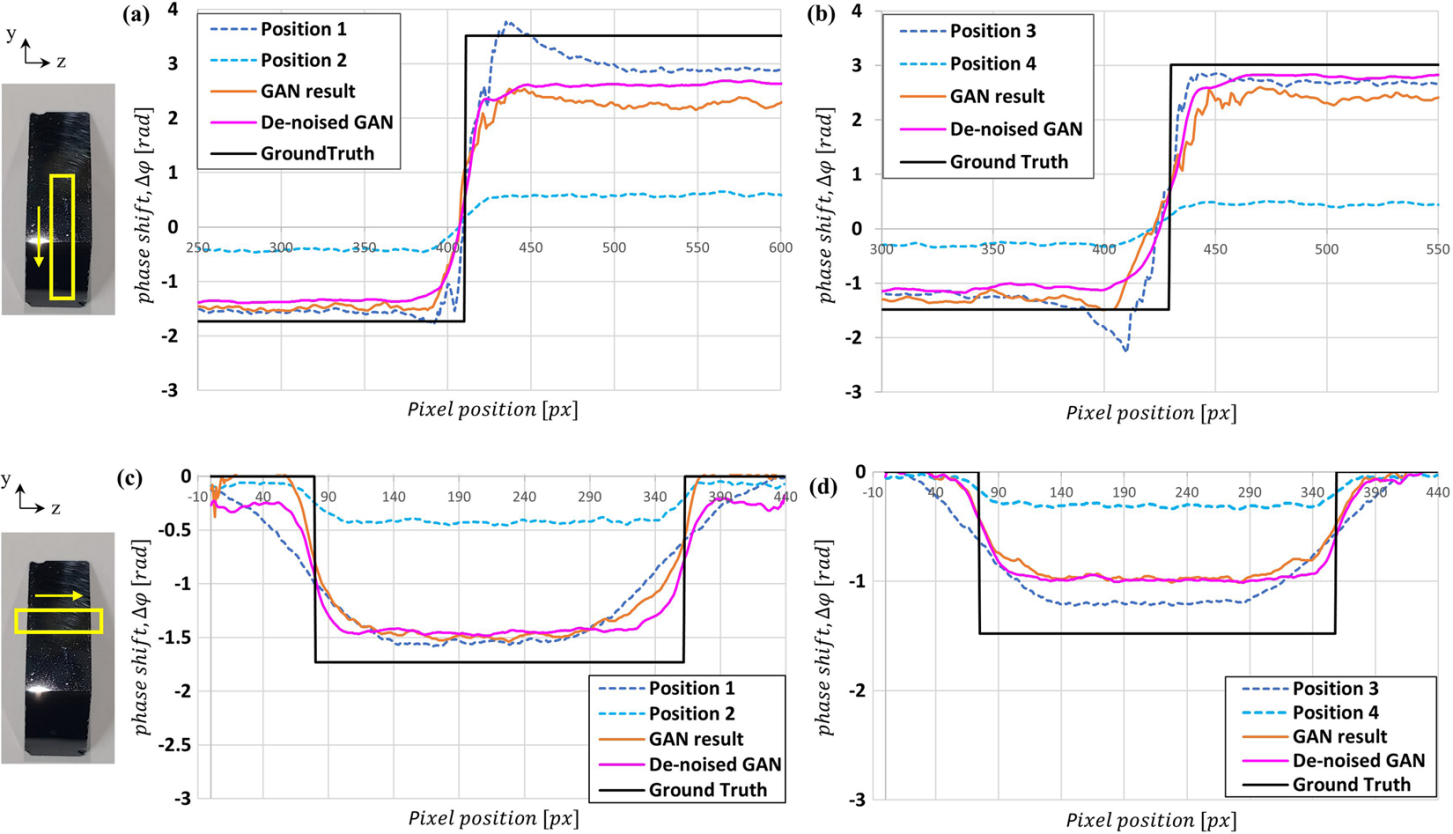
The longitudinal profiles of the images in Fig. 4(a,b) are shown in (**a**,**b**), respectively, and the transverse profiles are shown in (**c**,**d**). The profiles are the averaged ones in the yellow rectangle areas marked on the photos on the left. The solid profiles of ‘GAN result’ and ‘De-nosed GAN’ represent the compensation of spatial resolution and sensitivity by our 2-input GAN.

**Figure 6:**
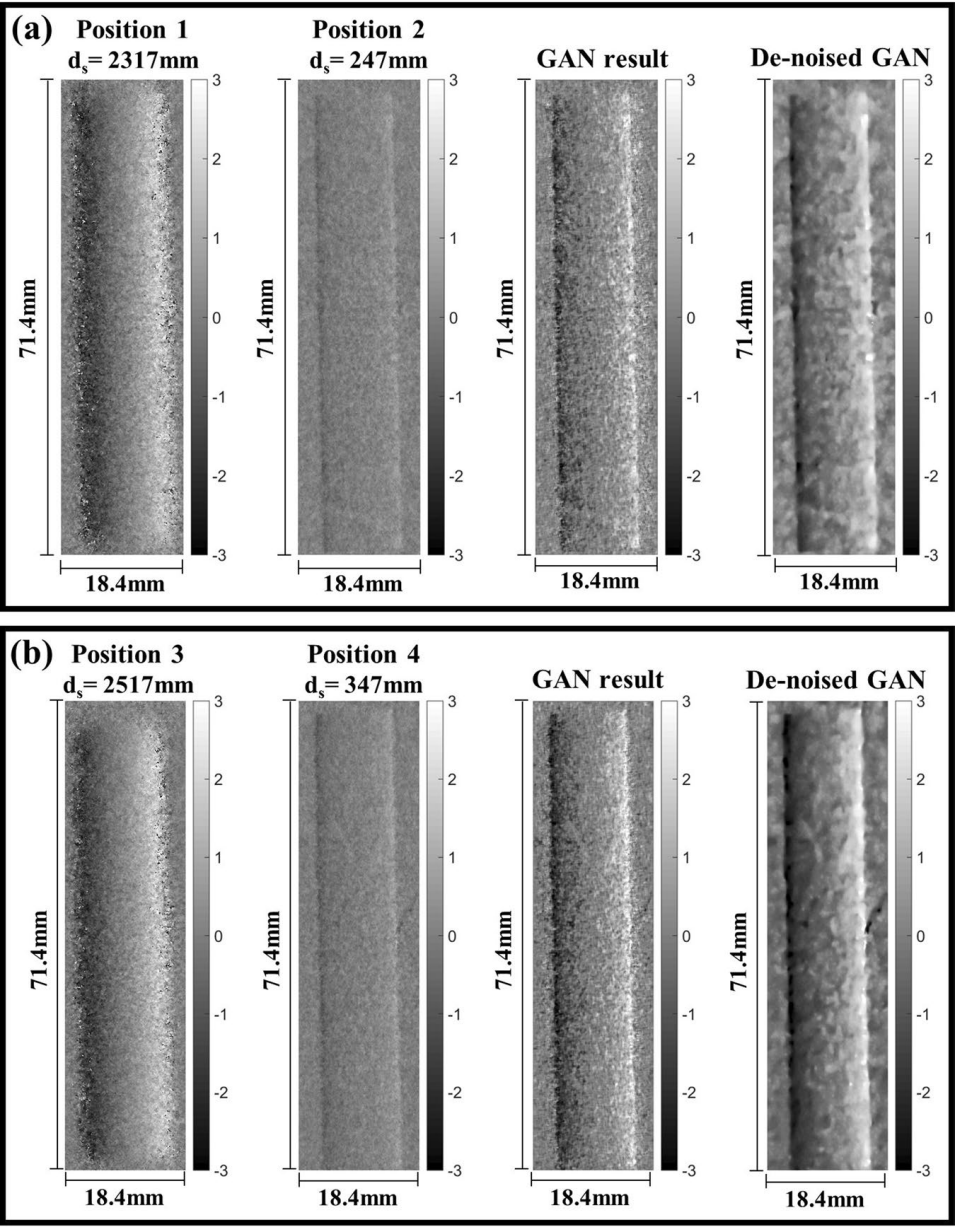
Two different input pairs illustrate the results of our study. (**a,b**) show the two input images and the corrected results of an Al cylinder. In (**a**), the sample positions(d_s_) are 4230 mm and 630 mm, and in (**b**), those are 3630 mm and 430 mm. Position 1 and 3 represent high-sensitivity images, and position 2 and 4 represent high-resolution images. The corrected results are shown as ‘GAN result’ and ‘De-noised GAN’.

**Figure 7:**
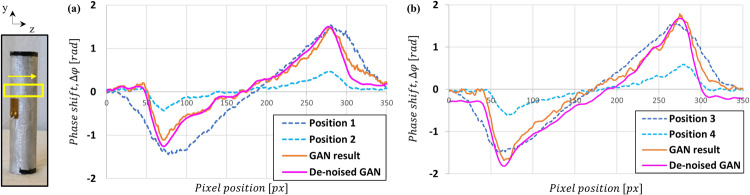
Transverse profiles of the (**a**,**b**) sample images at the Fig. 4. The profiles represent compensation of the resolution and sensitivity by the deep learning technique.

**Figure 8:**
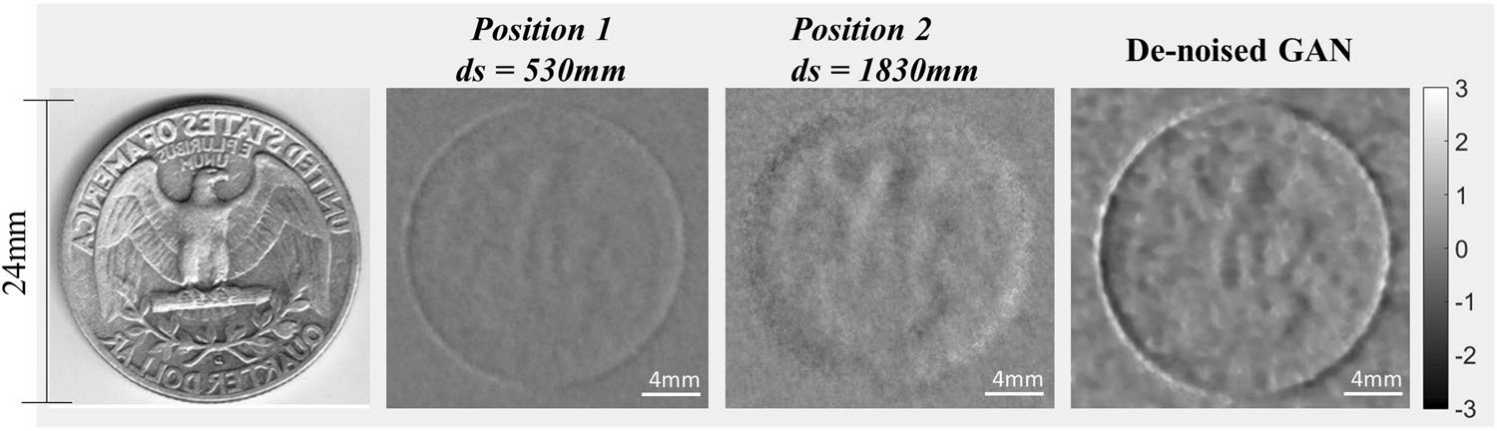
The experiment result of a quarter dollar. The experiment images were acquired in the two different sample position as inputs. The De-noised GAN well combine the two inputs. The inside of the quarter dollar was not clearly recovered, but the validity of the GAN as a tool for image registration is reliably confirmed.

In the neutron measurements, differential phase contrast images of a sample were obtained at the sample position, measured by *d*_*s*_ from the analyzer grating as shown in Fig. 1. The sample was measured at two sample positions for ‘high sensitivity and low resolution’ and ‘low sensitivity and high resolution’ images. The two measurements were used for inputs of the GAN and the results of a Si wedge, an Al cylindrical sample and quarter dollar are shown in Figs. 4–8. The Si wedge has an angle of 27° on one side and 46° on the other. In Figs. 4 and 6, the two inputs obtained by different positions used in the experiments, and their corrected images by the GAN are marked as ‘GAN result’ and ‘De-noised GAN’. Figures 5 and 7 show the comparative profiles of the inputs and results. The wedge sample was measured with the grating parameters of Set 1 in Table 1, and the two sample positions for ‘high sensitivity and low resolution’ and ‘low sensitivity and high resolution’ were chosen as 4230 mm and 630 mm for the inputs in the first row and 3630 mm and 430 mm for those in the second row in Fig. 4. In Fig. 4, the images in the column “GAN result” represent the combined image from our model and it demonstrates that the resolution and the sensitivity have been improved. However, the GAN results show incomplete recovery due to statistical noise and partial distortions in the images. To obtain better performance, we retrained our model by adjusting the noise level of the dataset. The results are shown in the De-noised GAN column in Fig. 4. Figure 5 shows the profiles along the vertical direction, which is the y-axis, and horizontal direction, which is the z-axis, of the wedge. To quantify the performance of the two-input channel GAN, we considered three factors: sensitivity, resolution and image noise. To this end, angular-sensitivity, full width at half maximum (FWHM), and signal-to-noise ratio (SNR) were obtained from the positional images of the Si wedge and the corrected images by GAN.

**Table 1:** Parameters of the neutron grating interferometer.

Design Wavelength			0.44 nm
Focal Spot size			13 mm
Set 1	Talbot order	3rd	
	Inter-grating distance	G0 − G1(L)	4,260 mm
		G1 − G2(D)	4,260 mm
	Period of gratings	p(p_0_ = p_1_ = p_2_)	50 μm
Set 2	Talbot order	1^st^	
	Inter-grating distance	G0 − G1(L)	3,636 mm
		G1 − G2(D)	3,636 mm
	Period of gratings	p(p_0_ = p_1_ = p_2_)	80 μm
Height of gratings		h_0_	100 μm (Gadox)
		h_1_	34.39 μm (Silicon)
		h_2_	100 μm (Gadox)

The ratio of the measured phase shift, *Δφ*, to the refraction angle, *α*, is a measure of the angular sensitivity^3^ with *S* = *Δφ/2πα*. The *α* measured by grating interferometer is derived by Donath *et al*.^3^ and interacted with neutron is derived by Kim *et al*.^7^ and reminded at Eq. (1).

1 $$|\alpha |=\frac{\lambda }{2\pi }\frac{\varDelta \phi }{\varDelta x}=\frac{{\lambda }^{2}}{2\pi }\frac{t}{\varDelta x}N{b}_{c}$$

where λ is the wavelength, *Δϕ* is the phase shift of neutron grating interferometer, *Δx* is the displacement of the sample in the y-direction in Fig. 1, *t* is the sample thickness in the x-direction, *N* is the atomic density, and *b*_*c*_ is the neutron scattering length. So, the *α* caused by one side of the Si wedge with an angle of 27° is 3.3 ± 0.2 µrad for a neutron wavelength of 0.44 nm, and the *Δφ* is the measured phase shift of the Talbot self-image. The *Δφ* was determined by measuring mean values in the image.

Also, the FWHM of the line spread function at the edge of the Si wedge was measured to compare the spatial resolution of each image. The SNR was obtained by dividing the standard deviation by the mean value of the same region from the images^33^. The results from the experimental images are reported in Table 2 and show the geometrical trade-off issues in the grating interferometer. However, the result obtained from the corrected images shown in Table 3 demonstrates that both input images are well combined by GAN. A second set of measurements were collected for an Al cylinder sample, and the grating configuration in this case corresponds to Set 2 in Table 3. Two pairs of measurement positions were chosen as 2317 mm and 247 mm for the first row and 2517 mm and 347 mm for the second row. In Fig. 6, the “GAN result” column shows the processed results from the input pairs of each row and the “De-noised GAN” column shows the results after training with more representative image noise in the training dataset. Figure 7 shows the averaged horizontal profiles of the (a) and (b) sample images in Fig. 6. The “GAN result” and “De-noised GAN” both show enhanced contrast and resolution but with different noise characteristics.

**Table 2:** Angular-sensitivity, FWHM, and SNR measured by Si wedge images.

	(a)		(b)	
***d***_***s***_ **[*mm*]**	4230	630	3630	430
***Angular*-*Sensitivity***	129,259	20,907	67,910	15,129
**FWHM [*mm*]**	4.26	0.82	3.65	0.77
***SNR***	29.6	5.5	16.3	4.1

**Table 3:** Angular-sensitivity, FWHM, and SNR measured by corrected images by deep learning techniques.

		(a)	(b)
***Angular-Sensitivity***	*GAN result*	96,982	72,716
	*De-noised GAN*	85,880	78,932
**FWHM** ***[mm]***	*GAN result*	0.77	0.72
	*De-noised GAN*	1.13	1.08
***SNR***	*GAN result*	8.4	5.1
	*De-noised GAN*	30	18.4

The results show numerically that the sensitivity, resolution, and image noise are well improved.

To evaluate a more complex shape, a quarter dollar coin was used as sample in geometry of Set 1 in Table 1, with sample positions of 530 mm and 1830 mm. Figure 8 shows the acquired images and the results of the De-noised GAN. Unlike the previous results, the coin image was not perfectly recovered except boundary, which we attributed to the lack of accurate shape information in both inputs. Despite this incomplete result, the image registration of two input images using GAN shows promise for further study and refinement.

## Discussion

According to the analysis of the sensitivity of the Talbot-Lau grating interferometer, the symmetrical Talbot-Lau grating interferometer can obtain the highest sensitivity for a fixed total system length. However, for neutron sources and conventional x-ray tubes, the increased distance between the sample and detector causes strong geometric blurring, and Kim *et al*.^7,8^ clearly showed the trade-off issue between the resolution and sensitivity. Since the blurring scale that occurs in the symmetrical geometry is very high, especially for a neutron beam line, the resolution improvement is quite important. In addition, nonuniform image distortion, where the phase shift rapidly changes in the high-sensitivity image, is also an issue.

**Figure 9:**
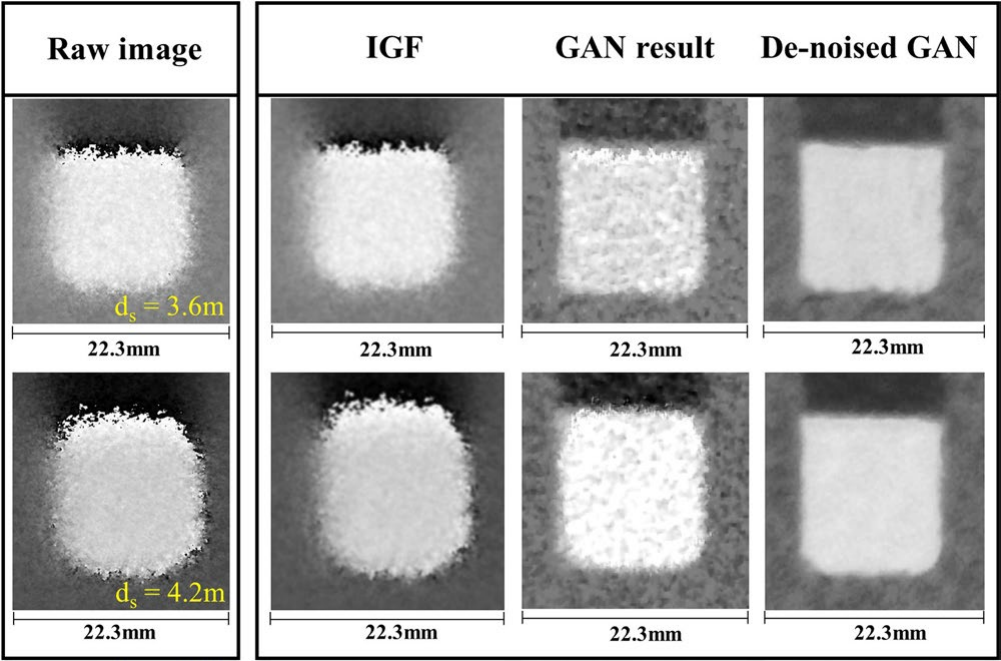
Comparison the results from the IGF and our model. Two-input based results are shown in GAN result and De-noised result column. When *d*_*s*_ is increased which is close to the phase grating G1, the image distortion occurs. IGF is not effective to solve this phenomenon, whereas our model restores more clearly.

In this study, we have applied a GAN algorithm for phase-contrast imaging to improve the phase-sensitivity and spatial resolution. We have constructed a two-input, very deep, residual-based GAN and successfully restored high resolution and high sensitivity images by setting the highest resolution image and the highest sensitivity image that can be obtained from a given system. The images used in our study were numerically analyzed by three factors: angular-sensitivity, FWHM, and SNR. As a result, we have verified that GAN is efficient to extract only the desired information from the two input images. To further place our work in context, in Fig. 9 we compare our 2-input GAN to an image guided filter (IGF)^34^ that corrects a blurry image using a clear image as a guide. The experimental image with very high sensitivity shows a kind of distortion when *ds* is increased. In this case, the IGF is not effective to restore resolution, whereas our GAN result clearly restored resolution. In addition, after training with a dataset whose noise more closely matched that of the raw images, is even more effective in restoring a clear image. Therefore, our study demonstrates the ability to extract both high resolution and high sensitivity information from the input images.

In future studies, we hope to reveal the limitations and supplements of our 2-input GAN as follows.

First, our model requires at least two images acquired at two different sample positions resulting in longer exposure times. The longer exposure time may be a disadvantage in medical applications, but the method we presented has applicability for research in which is a more accurate estimate of the phase gradient is required.

Second, two results using GAN are presented as “GAN result” and “de-noised GAN” in our study. The “GAN” result in our paper significantly shows our goal. We were able to get a good result by merging the resolution and sensitivity properly. Going one step further, however, we wanted to achieve better image by reducing noise level in terms of image processing through neural network. To do so, we proposed two idea. One of the two additional studies was presented as ‘De-noised GAN’, and the other idea is to analyze the neutron quantum noise in the NIST facility. Using a noise model more accurately reflecting the characteristics of NG6 bema line, we could further enhance signal-to-noise ratio of the corrected image. Through this, it is judged that the part of additional research should be developed in the form of additional papers.

Third, two input images were manually registered to apply our GAN model. Due to the minute misalignment of the hardware, images were not perfectly aligned. Despite the minor discrepancies, GAN corrected resolution and sensitivity from the two input images. Looking for ways to apply these subtle inconsistencies to learning will yield more accurate results.

Fourth, our GAN was trained with pseudo-phase contrast images, not images obtained from neutron grating interferometers. This is because an ideal image, that is, a high-resolution and high-sensitivity phase contrast image is required to train the network. However, it is challenging to experimentally acquire neutron phase contrast images with high-resolution and high-sensitivity information. In other words, since it is almost impossible to generate ground-truth, it was inevitably simulated with DIV2K. And that is the starting point of this paper. Since the GAN structure of this study is not a quantitative link between a phase shift value and a label like classification task, we considered that the quantitative refraction angle indices are not necessary. The core idea of our thesis is to extract the optimal result by combining of two different images properly. Since the input images are an experimental image, both images have diffraction information generated by a real neutron grating interferometer. We are convinced that a trained computer is just combining information from these two images, not a new price. Therefore, we believe that the reconstructed result image well reflects the neutron grating interferometer image.

In further studies such as phase unwrapping and phase information analysis, however, more realistic phase contrast images should be created for training.

Fifth, the Fig. 8 shows the significant result of our paper. The result of the “De-noised GAN” has both sharpness of position 1 and contrast information of position 2. It claims to satisfy the hypothesis that NN, the core idea of this paper, can match the optimal result from two different input images. Nevertheless, the Fig. 8 might not be seen the perfect coin. It is because the experimental image itself is incomplete^7^. As can be seen from the input images in Fig. 8, it is challenging to completely restore the coin due to the limitations of the experimental image itself.

Lastly, in the future, we would like to assess whether the two-input model has sufficient resolution and sensitivity to detect a minute crack generated in the sample. We speculate that we will need to construct an additional network for image segmentation to detect these fine cracks.

## Conclusion

Since the grating interferometer system provides three kinds of contrast images, which are absorption contrast, phase contrast, and dark-field contrast, our 2 input GAN increases the chance to detect a low contrast signal by combining with another contrast modality. Moreover, in the phase contrast image, one has the capability to adjust the phase contrast sensitivity based on the sample position. The phase contrast sensitivity increases as the sample gets closer to the phase grating, and the maximum sensitivity of a grating interferometer is highest when it is designed in symmetric geometry given the same source-to-analyzer grating distance. However, since the sample must be positioned closer to the phase grating for the highest sensitivity, the distance between the sample to the detector increases and we cannot avoid geometric blur. Hence, a trade-off problem with resolution and sensitivity becomes an important issue.

We hypothesized that the phase contrast images acquired at two positions between the phase grating and the analyzer grating contain abundant resolution and contrast information, and a deep learning method combining them helps generate a high phase-sensitive and high spatial-resolution image. We have trained a neural network using simulated phase contrast data pairs of “high sensitivity and low resolution” and “low sensitivity and high resolution” which can be acquired at two different positions between the phase contrast grating and the analyzer grating. We have applied the trained network to the neutron phase contrast images of a wedge, a cylinder sample, and a quarter dollar and demonstrated its effectiveness. Two representative image results with different de-nosing conditions and their profiles were shown for each sample cases. They clearly show that our GAN model significantly improves the sensitivity and resolution.

We have seen that it is possible to reconstruct a phase contrast image with both high phase-sensitive and high resolution using phase contrast image measurements at multiple positions in a grating interferometer using the GAN. This trade-off issue can often be met in a variety of other imaging modalities such as polychromatic far-field interferometer (PFI), single grid phase contrast imaging, super resolution phase contrast imaging, CT-MRI, PET-CT, and so on. Our model implemented in this study is expected to be similarly applicable to them as well to improve their sensitivity, spatial resolution, or other high dimensional images. Our results in this study is a demonstration of its initial feasibility and we expect further enhancement will be made in the near future.

## Methods

### Design of the high sensitivity Talbot-Lau interferometer

To design the high-sensitivity Talbot-Lau interferometer, we use the equation of a maximum sensitivity derived by Tilman Donath^3^:

2 $${S}_{max}=S({l}_{s}\approx l)=\frac{d}{{p}_{2}}=\frac{d}{M}\frac{1}{{p}_{1,e}},$$

where *p*_2_ is the period of G2 grating, *p*_*1,e*_ the effective period of grating G1, and M the geometrical magnification described by (*l* + *d*)/*l*. In the cone beam geometry, the *p*_*1, e*_ is described as the term of Talbot distance:

3 $${{p}_{1,e}}^{2}=\frac{2\lambda }{n}\frac{{d}_{n}}{M},$$

where *d*_*n*_ is Talbot distance in the cone beam geometry, *n* integer number represented by Talbot order, and λ wavelength. Now, the *S*_*max*_ is transformed by substituting Eq. (3) into Eq. (2):

4 $${S}_{max}=\frac{d}{M}\sqrt{\frac{nM}{2\lambda {d}_{n}}}.$$

Interference fringe behind G1 occurrs when the *d* is same as *d*_*n*_. Among the various designs of the grating interferometer, the phase-sensitivity according to the *l* is derived as:

5 $${S}_{{\max }}(l)=\sqrt{\frac{ndl}{2\lambda (l+d)}}.$$

**Figure 10:**
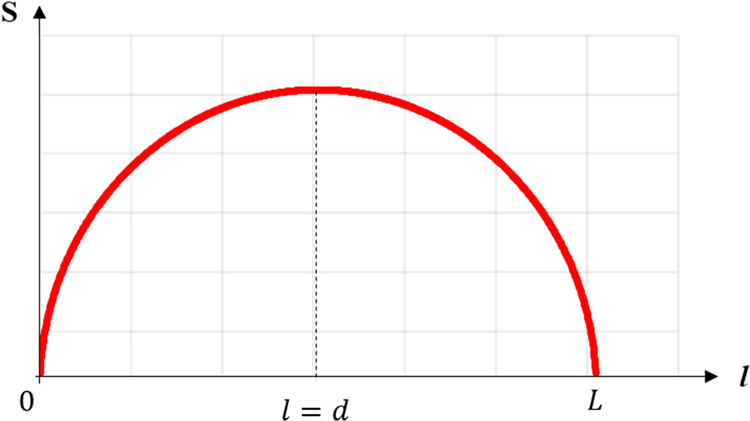
Sensitivity curve. The red line describes sensitivity along the grating G1 position. In the total length *L*, symmetric geometry (*l* = *d*) shows the highest sensitivity.

At fixed system length where *l* + *d* is a constant, Eq. (5) shows that the phase-sensitivity is proportional to the square root of *ld*. Therefore, Fig. 10 shows that among the interferometer geometries, the image with the highest phase-sensitivity is obtained in the symmetric geometry with the same distance between *l* and *d*.

### Hardware setup

**Figure 11:**
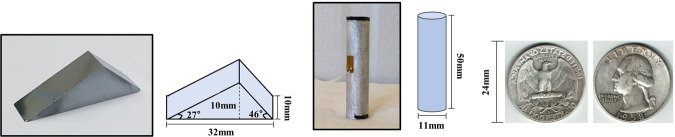
Silicon wedge (left), aluminum cylinder (middle), and quarter dollar (right) samples.

In this study, we used two sets of neutron gratings to acquire images^8^. The images were obtained at the cold neutron imaging beam line NG6 at the NIST Center for Neutron Research (NCNR)^35^. The source is polychromatic, and the mean energy wavelength is 0.44 nm. For one of the sets, all gratings have the same period of 50 μm; *l* = *d* = 4,260 mm; the Talbot order is 3. In the second set, all the gratings have the same period of 80 μm; and *l* = *d* = 3,636 mm; the Talbot order is 1. The detailed parameters are summarized in Table 1. This paper uses differential phase contrast images acquired through phase stepping in a direction perpendicular to the grating lines. The total number of steps is 8 and the acquired images are processed through Fourier analysis^36^. A single image per step was acquired over a few seconds of exposure time, and a total of six images were integrated through a median filter. The detector in the experiment is an Andor Neo, a scientific complementary metal-oxide semiconductor, camera viewing a 200 μm thick LiF:ZnS scintillator screen through a 50 mm lens yielding an effective pixel pitch of 51.35 μm. (Certain trade names and company products are mentioned in the text or identified in an illustration in order to adequately specify the experimental procedure and equipment used. In no case does such identification imply recommendation or endorsement by the National Institute of Standards and Technology, nor does it imply that the products are necessarily the best available for the purpose.) (scientific complementary metal-oxide semiconductor) camera viewing a 200 µm thick LiF:ZnS scintillator screen through a 50 mm lens yielding an effective pixel pitch of 51.35 μm. Figure 11 shows the silicon (Si) wedge, aluminum (Al) cylinder, and quarter dollar samples for the experiment. The Si wedge has different triangular prism angles from both corners to vertexes, so the different phase-shift for each side will be measured. The Al cylinder will measure linear gradient of the phase-shift along the surface. The quarter dollar is used as demo sample for low sensitivity as well as complex shape.

### Data

**Figure 12:**
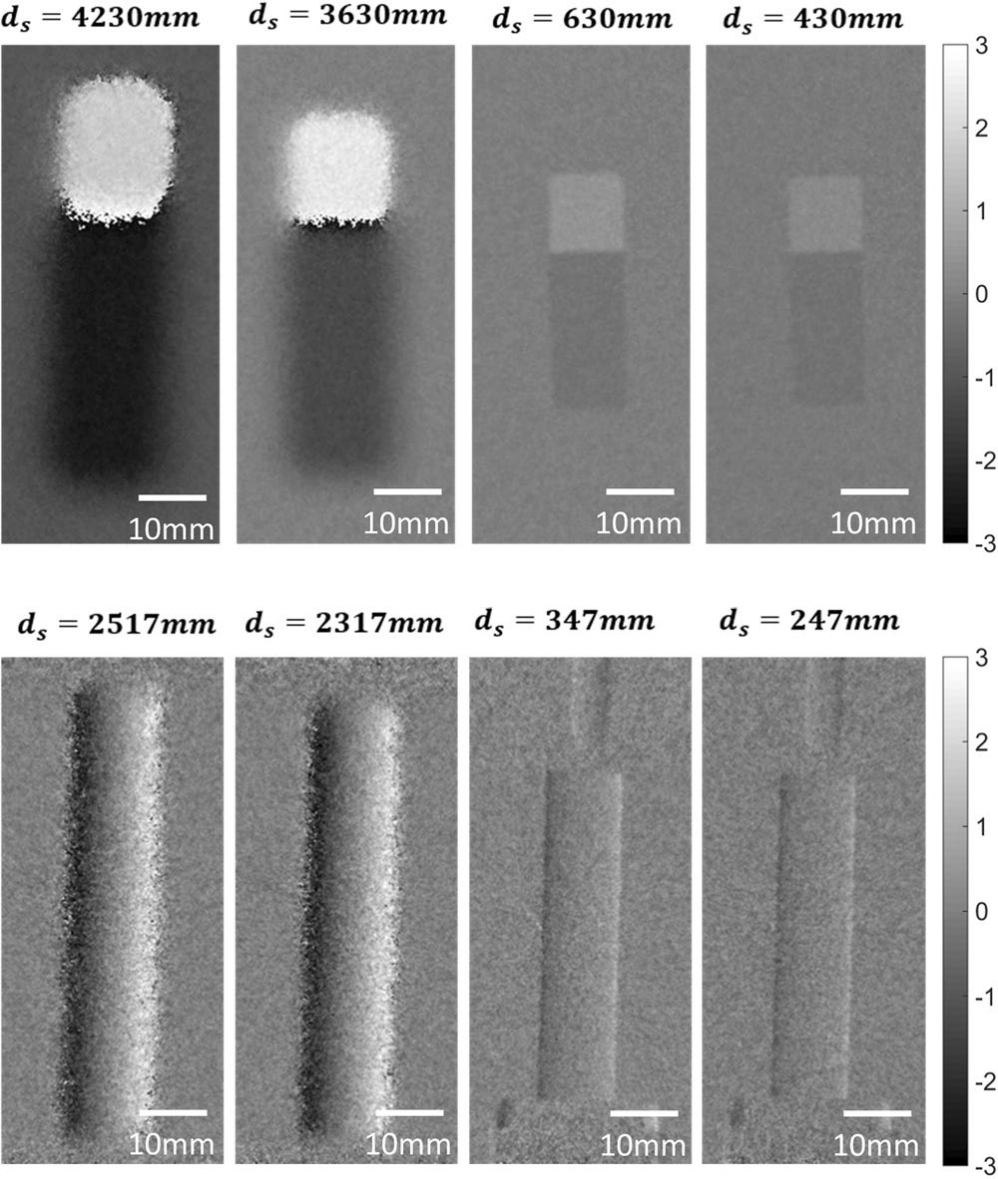
Differential phase contrast images of silicon wedge acquired by set 1 (top) and aluminum cylinder acquired by set 2 (bottom). Each sample is obtained at different *d*_*s*_. The longer distance of the *d*_*s*_ presents the higher sensitivity and lower spatial resolution.

Figure 12 shows differential phase contrast images of the Si wedge and Al cylinder with same gray scale. Images of the Si wedge and Al cylinder were acquired by set 1 and set 2, respectively. Each image was obtained at different *d*_*s*_ which was achieved by a motorized stage. In Fig. 12, the sensitivity is linearly increased when the sample is close to the phase grating G1. Thus, in the same system, the phase sensitivity ratio is almost similar to the *d*_*s*_ ratio. In addition, the degradation of the resolution is shown as image blurring and distortion. To improve the image clarity by GAN, we additionally consider image magnification as well. Since the image magnification depends on the distance *d*_*s*_, we can simply match the alignment of two images.

### Creation of training dataset

A large amount of high-sensitivity low-resolution image and low-sensitivity high-resolution image pairs are required to train neural network. Instead of using real differential phase contrast images, this study created a large dataset that simulates phase contrast image by converting color images to 32-bit gray scale images. The DIV2K dataset^37,38^ were thus prepared and then the gray scales were also normalized from −π to +π to mimic the differential phase contrast images. From these converted images, two images with different sensitivity and resolution are created and converted image itself was used as the ground-truth. Since the phase sensitivity in the measurements is linearly proportional to d_s_, the training images are simply scaled by a liner constant. The high-sensitivity low-resolution image was realized by convolution with a Gaussian smoothing filter in the converted image to express unsharpness. Finally, a random gaussian noise was added to realize the Poisson noise of the neutron beam. The noise level was determined by measuring the standard deviation of experimental background image in our system. A total of 890 iamges were converted to normalized gray scale images, so 890 image pairs were created for training input. Due to a large size of each picture size of larger than 2000 × 2000 pixels, however, we made 128 × 128 patches. So, more than 200,000 training datasets were created.

### Deep learning Network

We designed a deep residual network-based GAN^9^, and the model was implemented by Tensorflow. In general, a GAN network consists of two different networks. One is a generator, and another is a discriminator. The generator network is trained to generate images similar to the ground-truth, and the discriminator network is trained to determine whether the ground-truth and generated image from the generator are equal or different.

**Figure 13:**
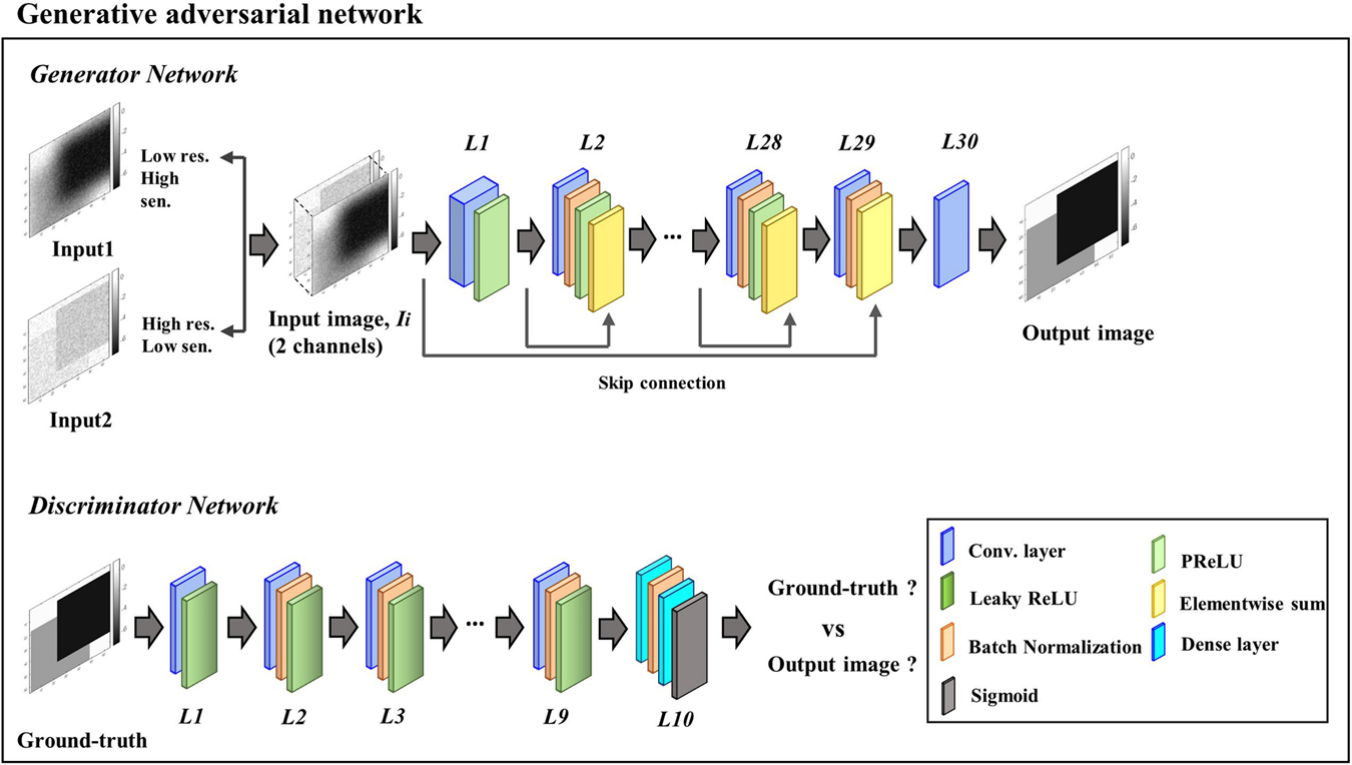
Architecture of the deep residual-based GAN. In our study, we set input channel to 2 to insert two different input images.

To build a generator, variables, known as placeholders in Tensorflow, are first defined with size of 128 × 128 × 2. In each trial, many training datasets go through a convolution operation process with these placeholder variables. The variables are connected through convolution layers where each convolution layer has a 3 × 3 kernel and the number of kernels is 64 in our model. A batch-normalization layer was applied between each convolution layer to reduce the internal covariate shift issue^39,40^. The output of a convolution layer is then passed through a rectified linear unit (ReLU) function known as one of the activation functions^41^. The ReLU function converts complex convolution values into a simple logistic regression model by transferring zero when the input is negative and equal value when input is zero or positive value. Total 20 pairs of convolution, batch normalization, and ReLU layers were created for the generator. These layer pairs are connected based on the residual blocks and skip-connections^42^ shown at Fig. 13. The output image is now updated by comparing with ground-truth, and the optimization is conducted by minimizing the mean square error (MSE) between the output image and ground-truth. The MSE in our model is defined as generator loss.

To build a discriminator, variables known as placeholders in Tensorflow are defined with size of 128 × 128 × 1. A total of 10 pairs of convolution, batch normalization, and leaky ReLU layers were then created and directly connected to the variables. Each convolution layer has 3 × 3 kernel and the number of kernels varies from 64 to 512. The leaky ReLU^43^ transfers a small positive gradient rather than zero when the input is negative value and equal value when input is zero or positive value. In the last layer, we additionally inserted a dense layer that fully connects a large amount of convolution values into one layer. The fully connected layer is converted to a probability map through the logits function.

To optimize the discriminator, two logits of the generator output and ground-truth are created, respectively. The generator output is defined as fake image, and cross entropy is calculated by its logits approaching zero. The ground-truth is defined as real image, and cross entropy is calculated by its logits approaching one. The sum of two cross entropies in our model are defined as discriminator loss. Our network is now optimized by minimizing both generator loss and discriminator loss. Unlike the general architecture, in this study, the first layer and input-channels were set to 2. Therefore, we demonstrate how the neural network combines image sensitivity and resolution from each input image.

## References

[1] Pfeiffer F (2008). Hard-X-ray dark-field imaging using a grating interferometer. Nat Mater.

[2] Pfeiffer F, Weitkamp T, Bunk O, David C (2006). Phase retrieval and differential phase-contrast imaging with low-brilliance X-ray sources. Nat Phys.

[3] Donath, T. *et al*. Inverse geometry for grating-based x-ray phase-contrast imaging. *J Appl Phys* **106** (2009).

[4] Birnbacher, L. *et al*. Experimental Realisation of High-sensitivity Laboratory X-ray Grating-based Phase-contrast Computed Tomography. *Sci Rep-Uk* **6** (2016). 10.1038/srep24022 PMC4819174 27040492

[5] Lee SW (2017). High-Resolution X-Ray Phase-Contrast Imaging with a Grating Interferometer. J Korean Phys Soc.

[6] Engelhardt, M. *et al*. High-resolution differential phase contrast imaging using a magnifying projection geometry with a microfocus x-ray source. *Appl Phys Lett* **90** (2007). 10.1063/1.2786273 17902955

[7] Kim, Y., Kim, J., Kim, D., Hussey, D. S. & Lee, S. W. Characterization of the phase sensitivity, visibility, and resolution in a symmetric neutron grating interferometer. *Rev Sci Instrum* **90** (2019). 10.1063/1.5089588 31370431

[8] Kim, Y., Kim, J., Kim, D., Hussey, D. S. & Lee, S. W. Feasibility evaluation of a neutron grating interferometer with an analyzer grating based on a structured scintillator. *Rev Sci Instrum* **89** (2018). 10.1063/1.5009702 PMC8628295 29604735

[9] Ledig, C. *et al*. In *Proceedings of the IEEE conference on computer vision and pattern recognition*. 4681–4690.

[10] Zhou FQ, Li XJ, Li ZX (2018). High-frequency details enhancing DenseNet for super-resolution. Neurocomputing.

[11] Liu H, Han JG, Hou SD, Shao L, Ruan Y (2018). Single image super-resolution using a deep encoder-decoder symmetrical network with iterative back projection. Neurocomputing.

[12] Zeng K, Yu J, Wang RX, Li CH, Tao DC (2017). Coupled Deep Autoencoder for Single Image Super-Resolution. Ieee T Cybernetics.

[13] Sun YW, Li LT, Cong P, Wang ZT, Guo XJ (2017). Enhancement of digital radiography image quality using a convolutional neural network. J X-Ray Sci Technol.

[14] Kim, J., Lee, J. K. & Lee, K. M. Accurate Image super-resolution using very deep convolutional networks. *IEEE Conference on Computer Vision and Pattern Recognition*, 1646–1654 (2016).

[15] Wang, Z., Liu, D., Yang, J., Han, W. & Huang, T. Deep Networks for Image Super-Resolution with Sparse Prior. *Proceedings of the IEEE International Conference on Computer Vision*, 370–378 (2015).

[16] Zhangyang Wang *et al* S. Huang. Self-Tuned Deep Super Resolution. *Proceedings of the Computer vision and Pattern Recognition Workshop on Deep Vision*, 1–8 (2015).

[17] Simonyan, K. & Zisserman, A. In *International Conference on Learning representations (ICLR)* (2015).

[18] Dong C, Loy CC, He K, Tang X (2015). Image Super-Resolution Using Deep Convolutional Networks. IEEE TRANSACTIONS ON PATTERN ANALYSIS AND MACHINE INTELLIGENCE.

[19] Yasaka K, Akai H, Kunimatsu A, Abe O, Kiryu S (2018). Liver Fibrosis: Deep Convolutional Neural Network for Staging by Using Gadoxetic Acid-enhanced Hepatobiliary Phase MR Images. Radiology.

[20] Tiulpin, A., Thevenot, J., Rahtu, E., Lehenkari, P. & Saarakkala, S. Automatic Knee Osteoarthritis Diagnosis from Plain Radiographs: A Deep Learning-Based Approach. *Sci Rep-Uk* **8** (2018). 10.1038/s41598-018-20132-7 PMC5789045 29379060

[21] Sors A, Bonnet S, Mirek S, Vercueil L, Payen JF (2018). A convolutional neural network for sleep stage scoring from raw single-channel EEG. Biomed Signal Proces.

[22] Fong, R. C., Scheirer, W. J. & Cox, D. D. Using human brain activity to guide machine learning. *Sci Rep-Uk* **8** (2018). 10.1038/s41598-018-23618-6 PMC5876362 29599461

[23] Diederich, B., Wartmann, R., Schadwinkel, H. & Heintzmann, R. Using machine-learning to optimize phase contrast in a low-cost cellphone microscope. *Plos One* **13** (2018). 10.1371/journal.pone.0192937 PMC5832211 29494620

[24] Chartsias A, Joyce T, Giuffrida MV, Tsaftaris SA (2018). Multimodal MR Synthesis via Modality-Invariant Latent Representation. IEEE T Med Imaging.

[25] Wang G (2016). A Perspective on Deep Imaging. IEEE Access.

[26] Litjens, G. *et al*. Deep learning as a tool for increased accuracy and efficiency of histopathological diagnosis. *Sci Rep-Uk* **6** (2016). 10.1038/srep26286 PMC4876324 27212078

[27] Zhang, H. *et al*. Image Prediction for Limited-angle Tomography via Deep Learning with Convolutional Neural Network. *arXiv preprint* arXiv:1607.08707 (2016).

[28] Qayyum, A., Saad, N. M., Kamel, N. & Malik, A. S. Deep convolutional neural network processing of aerial stereo imagery to monitor vulnerable zones near power lines. *J Appl Remote Sens* **12** (2018).

[29] An, Q. Z., Pan, Z. X. & You, H. J. Ship Detection in Gaofen-3 SAR Images Based on Sea Clutter Distribution Analysis and Deep Convolutional Neural Network. *Sensors-Basel* **18** (2018). 10.3390/s18020334 PMC5855143 29364194

[30] van Aarle W (2016). Fast and flexible X-ray tomography using the ASTRA toolbox. Opt Express.

[31] van Aarle W (2015). The ASTRA Toolbox: A platform for advanced algorithm development in electron tomography. Ultramicroscopy.

[32] Segars WP, Mahesh M, Beck TJ, Frey EC, Tsui BMW (2008). Realistic CT simulation using the 4D XCAT phantom. Med Phys.

[33] Tapiovaara MJ, Wagner RF (1993). Snr and Noise Measurements for Medical Imaging - Ia Practical Approach Based on Statistical Decision-Theory. Phys Med Biol.

[34] He, K., Sun, J. & Tang, X, Guided Image Filtering, IEEE transactions on pattern analysis and machine intelligence, **35**, 1397–1409 (2012). 10.1109/TPAMI.2012.213 23599054

[35] Hussey DS (2015). A New Cold Neutron Imaging Instrument at NIST. Physics Procedia.

[36] Bech, M. *X-ray imaging with a grating interferometer* PhD thesis, University of Copenhagen, Denmark, (2009).

[37] Timofte, R. & Agustsson, E. DIV2K dataset, https://data.vision.ee.ethz.ch/cvl/DIV2K/ (2017).

[38] Agustsson, E. & Timofte, R. In *Proceedings of the IEEE Conference on Computer Vision and Pattern Recognition Workshops*. 126–135.

[39] Liu MF (2018). Deep learning based on Batch Normalization for P300 signal detection. Neurocomputing.

[40] loffe, S. & Szegedy, C. In *Proceedings of the 32nd International Conference on Machine Learning, ICML* (2015).

[41] Ide, H. & Kurita, T. In *Improvement of learning for CNN with ReLU activation by sparse regularization.”* 2017 *International Joint Conference on Neural Networks (IJCNN)*, IEEE, (USA, 2017).

[42] Yamanaka J, Kuwashima S, Kurita T (2017). International Conference on Neural Information Processing.

[43] Mass, A. L., Hannun, A. Y. & Ng, A. Y. In *Proceedings of the 30th International Conference on Machine Learning*. 3.

