# Author Correction: Deep learning for high-resolution and high-sensitivity interferometric phase contrast imaging

**DOI:** 10.1038/s41598-021-88565-1

**Published:** 2021-04-19

**Authors:** Seho Lee, Ohsung Oh, Youngju Kim, Daeseung Kim, Daniel S. Hussey, Ge Wang, Seung Wook Lee

**Affiliations:** 1 School of Mechanical Engineering, Pusan National University, Busan, 46241 Republic of Korea; 2 Neutron Physics Group, National Institute of Standards and Technology, Gaithersburg, MD 20899 USA; 3 Department of Biomedical Engineering, Rensselaer Polytechnic Institute, Troy, NY 12180 USA

Correction to: *Scientific Reports* 10.1038/s41598-020-66690-7, published online 18 June 2020

The Acknowledgements section in this Article is incomplete.

“This work was supported by National R&D (Grant Nos. NRF-2017K1A3A7A09016309 and NRF-2019R1A2C1007491) and Radiation Technology R&D (Grant No. NRF-2017M2A2A6A01016849) program through the National Research Foundation of Korea funded by the Ministry of Science and ICT. DSH acknowledges support from the U.S. Department of Commerce, the NIST Radiation Physics Division, the Director’s office of NIST, and the NIST Center for Neutron Research.”

should read:

“This work was supported by National R&D (Grant Nos. NRF-2017K1A3A7A09016309 and NRF-2019R1A2C1007491, NRF-2020K1A3A7A09078093) and Radiation Technology R&D (Grant No. NRF-2017M2A2A6A01016849) program through the National Research Foundation of Korea funded by the Ministry of Science and ICT. DSH acknowledges support from the U.S. Department of Commerce, the NIST Radiation Physics Division, the Director’s office of NIST, and the NIST Center for Neutron Research.”

